# Large-scale semi-arid afforestation can enhance precipitation and carbon sequestration potential

**DOI:** 10.1038/s41598-018-19265-6

**Published:** 2018-01-17

**Authors:** Gil Yosef, Robert Walko, Roni Avisar, Fedor Tatarinov, Eyal Rotenberg, Dan Yakir

**Affiliations:** 1 Earth and Planetary Sciences, Weizmann Institute of Science, Rehovot, Israel; 2 Rosenstiel School of Marine and Atmospheric Science, University of Miami, Miami, Florida USA

## Abstract

Afforestation is an important approach to mitigate global warming. Its complex interactions with the climate system, however, makes it controversial. Afforestation is expected to be effective in the tropics where biogeochemical and biogeophysical effects act in concert; however, its potential in the large semi-arid regions remains insufficiently explored. Here, we use a Global Climate Model to provide a process-based demonstration that implementing measured characteristics of a successful semi-arid afforestation system (2000 ha, ~300 mm mean annual precipitation) over large areas (~200 million ha) of similar precipitation levels in the Sahel and North Australia leads to the weakening and shifting of regional low-level jets, enhancing moisture penetration and precipitation (+0.8 ± 0.1 mm d^−1^ over the Sahel and +0.4 ± 0.1 mm d^−1^ over North Australia), influencing areas larger than the original afforestation. These effects are associated with increasing root depth and surface roughness and with decreasing albedo. This results in enhanced evapotranspiration, surface cooling and the modification of the latitudinal temperature gradient. It is estimated that the carbon sequestration potential of such large-scale semi-arid afforestation can be on the order of ~10% of the global carbon sink of the land biosphere and would overwhelm any biogeophysical warming effects within ~6 years.

## Introduction

Afforestation is considered as climate change mitigation strategy^1–3^, but it is also associated with potential climate feedbacks^2,4^ and rarely considers the importance of semi-arid regions^5^. Attempts to explain the records that indicate the “greening of the Sahara” 6 to 9 thousand years ago^6^, showed that the “top-down” effects of changes in the earth-system that ultimately result in land cover changes, such as changes in the sea surface temperature (SSTs) of the Atlantic, Indian and Pacific Ocean basins, can be associated with changes in land-ocean circulation and teleconnection that influence moisture transport and local precipitation over semi-arid monsoon regions such as the Sahel^7,8^. The resulting changes in land cover can consequently lead to changes in surface temperature gradients and, in turn, to changes in the characteristics of local atmospheric circulation, such as the African easterly jet (AEJ) and the intensity of the monsoon westerly (MW) winds in regions such as the Sahel^9–11^. Similar processes to those described for the Sahel were also indicated for the Asian monsoon in Northern Australia^12,13^, including the development of the Australian Easterly Jet (AUSEJ).

In contrast to the “top-down” effects noted above, locally driven, “bottom-up” climatic effects triggered by first changing the land-cover in semi-arid regions, such as desertification in the Sahel, were demonstrated by the pioneering work of Charney^14^, Ornstein^11^ and others^15,16^. Inverse feedbacks also operated on re-forestation in such regions (e.g.^11,17–19^). While these pioneering studies dealt with some aspects of land cover and climate in the Sahel and similar regions, we make an effort in this study to addresses some of their limitations and provide a comprehensive and process-based perspective as the question of the potential contribution of afforestation to climate change mitigation becomes more urgent. For example, some of the early studies relied on low resolution models (of about 2.5° to 4° latitude and 3° to 5° longitude and less than 20 *σ* vertical levels), independent of global ocean effects, and with vegetation type inappropriate for semi-arid climate (e.g.^9,11,19^). Some studies were based on local perspective driven by local evaporation (e.g.^9,20,21^), or increases in turbulent kinetic energy^22^. In contrast, some studies tended to emphasize mainly the ocean teleconnection effects on precipitation regime in West Africa^11,15,23^. Additional perspectives were also reported, such as associated with deforestation outside the monsoon regions^24^, or afforestation in the northern mid-latitudes^25^.

At present, it is recognized that the effects of afforestation and other land-use changes on climate are often assessed through its biogeochemical (BGC) effect, such as carbon sequestration that influences atmospheric CO_2_ concentrations, and the direct effects on surface energy budget associated with biogeophysical (BGP) effects, such as reduced albedo, increased surface roughness and enhanced evapotranspiration^26^. The effectiveness of afforestation as a climate change mitigation strategy depends on the balance of the BGC and BGP effects and was shown to be most effective in the tropics, where water availability enhances carbon uptake and cloudiness, with the later balancing the reduced albedo of the forest surface (e.g.^2^). It has been argued, based on previous research, to be less effective or even counterproductive in other regions where carbon uptake is more limited, and its climatic effects are overwhelmed by the resulting reduction in albedo, leading to warming^1,4^. Note that other effects, such as the emission of volatiles that influence cloud formation, could also influence the overall effectiveness of afforestation^27^.

Here we use a comprehensive global and process-based approach to re-consider the idea that large-scale afforestation over semi-arid monsoon regions can be sustainable, improve local climatic conditions and provide a basis for large-scale carbon sequestration, and consequently also wood and food production. We carried out numerical experiments with a Global Circulation Model (GCM) to implement large-scale, semi-arid afforestation, based on the observed physical characteristics of a successful small-scale, semi-arid forest, which has been extensively monitored over the past 16 years, growing at the edge of the Negev desert with a mean annual precipitation of ~300 mm^17^. This study considerably extends and diverges from earlier sensitivity tests of short-term regional, coarser-resolution global, or based on artificial-irrigation, or un-realistic plant functional types studies (e.g.^2,11,19^).

### Forestation effects on precipitation and temperature in the afforested regions

**Figure 1:**
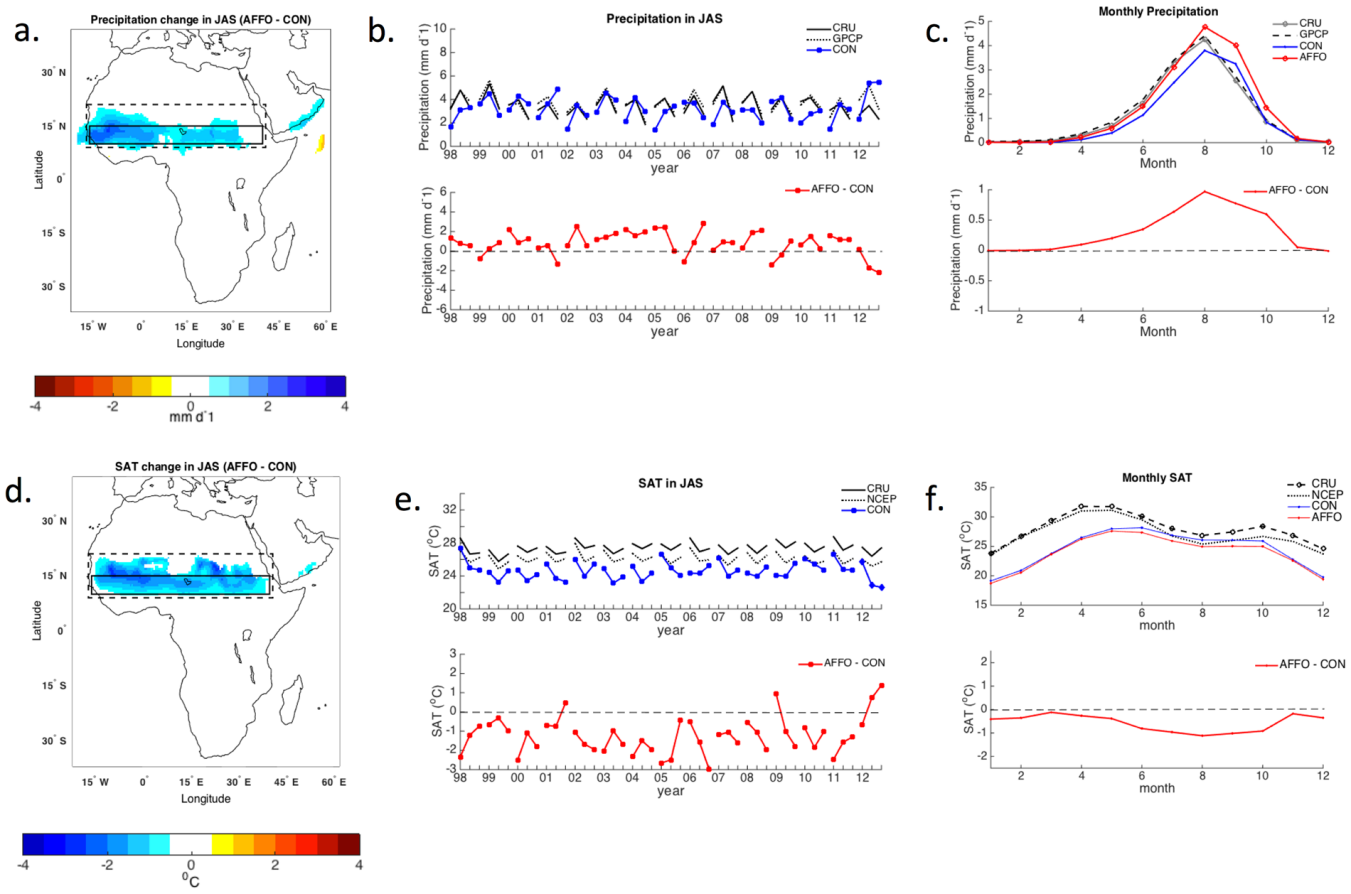
Observations (CRU, GPCP and NCEP) and model-simulated precipitation and surface-air-temperature (SAT) over Africa between the years 1998 and 2012. Precipitation: (**a**) Spatial patterns of observed seasonal mean of July-August-September (JAS) bias between afforestation (AFFO-2d-S) minus control (CON-2d) with 95% confidence level. The solid and dashed lines cover the afforested area [10-15°N, 16° W-40°E] and the footprint area, respectively [10°-20°N, 16°W-40°E]. (**b**) Time series of rainfall over the afforested area for consecutive JAS. (**c**) Seasonal evolution over the afforested area. The same for (d-e) SAT. Maps in this figure were created using Matlab R2014b (http://www.mathworks.com/).

A new ‘plant functional type’ (PFT) was defined for this study using the available physical characteristics of a successful semi-arid afforestation system in Yatir^17,18^. This PFT was used to replace all the land cover of the native vegetation, over the semi-arid experimental area in the Sahel and North Australia (see Methods). The results of the model simulations clearly indicated increase in precipitation and cooling of surface air temperature (SAT) over both the Sahel and North Australia. In the Sahel scenario, the results show a spatial pattern of anomalies stretching zonally across the afforested area and a footprint (area of influence) of the effects extending up to 20°N well beyond the afforested area, as shown in the 95% confidence maps in Fig. 1a,d. Monthly average precipitation over the Sahel region showed precipitation development around April and ending around November (Fig. 1c), with a maximum effect in the summertime, July-August-September (JAS), with a mean positive bias of **+**0.8 ± 0.1 mm d^−1^ (Fig. 1b,c). The afforestation effects on the regional surface temperature indicated cooler SAT year around, with the maximum change in summertime, and a mean change of −1.3 ± 0.1 °C (Fig. 1e,f). The afforestation effects on SAT were influenced by the increase in cloud cover and cold advection of wind from the ocean and the forest (see SI Fig. 6d-f), which extended into the southern parts of the Sahara Desert.

For both precipitation and SAT, the 15-year simulations showed robust results of consistently higher precipitation and lower SAT throughout the entire simulation period and across the inter-annual climate variations, including three significant El-Nino (i.e., 1997/8, 2002/3, 2009/10) and four La-Nina years (i.e., 1998/9, 1999/00, 2007/8, 2010/11). Comparing the longer and lower-resolution (15 years, 200 km) simulations to the short-term high-resolution simulations (3 years, 50 km) also indicated consistent results of increasing precipitation and cooler surface (with somewhat higher mean anomaly values in the high-resolution simulation; see SI Figs 3 and 4).

Similar results were observed in the afforestation simulations in North Australia, where in the summertime, precipitation increased on average by **+**0.4 ± 0.1 mm d^−1^, and SAT decreased by −0.9 ± 0.1 °C on average over the forested area. The North Australia results are not discussed in detail below but are reported in the SI (see SI Fig. 2). We show, however, that the results over semi-arid areas under the monsoon regime in both the Sahel and North Australia are associated with the same mechanisms; where surface cooling modifies the latitudinal temperature gradient, weakens and shifts the regional low-level jets, leading to enhancing moisture penetration and precipitation.

### Processes underlying the changes in precipitation and SAT

#### Surface Energy Partitioning

Changes in SAT were associated with an increase in the surface net radiation of **+**9.5 ± 1.4 W m^−2^ (see SI Table. 5). This was mainly due to changes in the long-wave thermal radiation balance: an increase in downward **(+**2.4 ± 0.8 W m^−2^) due to increase atmospheric moisture and cloudiness and a decrease in upward thermal radiation flux (−8 ± 0.9 W m^−2^) due to the lower surface temperature. Changes in short-wave radiation essentially balanced out, with the albedo effect increasing surface absorption **(+**14.8 ± 1.1 W m^−2^) and reduced incoming short-wave radiation due to increased atmospheric moisture and cloudiness (−15.2 ± 1.9 W m^−2^). The increase in total available energy combined with the increasing availability of soil moisture (initially due to increasing root depth and subsequently to increase in precipitation) were transformed into non-radiative surface fluxes, with a large increase in the latent heat flux^28^, LE **(+**16.9 ± 2.5 W m^−2^) and a small decrease in the sensible heat flux, H (−5.6 ± 0.9 W m^−2^). The small change in H likely reflected the balance between surface cooling and low canopy aerodynamic resistance. The resulting overall cooling of the surface led, in turn, to changes in the meridional gradient in SAT.

#### Changes in the regional atmosphere circulation

**Figure 2:**
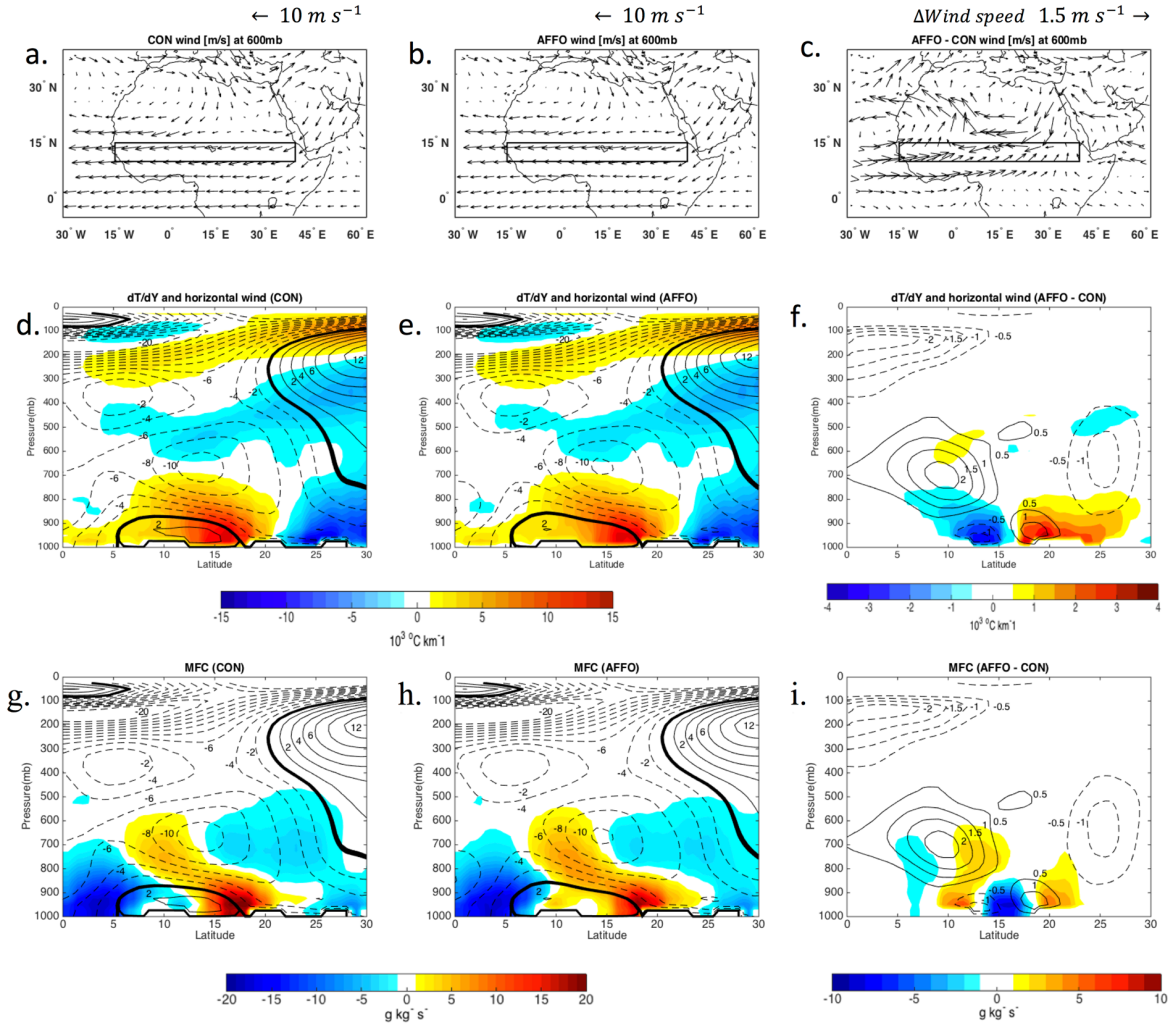
Simulated spatial patterns of wind at 600 mb (**a**–**c**), temperature gradients, dT/dy [10^3^ °C km^−1^] (**d**–**f**), and total moisture flux convergence (MFC) or divergence (negative values) [10^3^ g kg^−1^ s^−1^] with zonal wind [m s^−1^] (**g**–**i**). Seasonal mean values over Africa are for July. to September. for the simulation period 1998–2012, and zonally averaged (10°W-15°W). Left, middle and right panels represent control (CON-2d), afforestation (AFFO-2d-S) and bias between afforestation minus control, respectively. Solid lines and dash line represents easterly and westerly winds intensity respectively. Maps were created using Matlab R2014b (http://www.mathworks.com/).

The surface cooling over the forested area affected the meridional temperature gradient by decreasing it over the forested area and increasing it over the drier interface of the afforestation band (Fig. 2d–f). As expected, such changes in surface temperature gradients weakened the local low-level jet (the so called African-Easterly-Jet, AEJ), with a change of 2 m s^−1^ at its core and spatial displacement toward the drier edge of the forested band (Fig. 2a–c). This is likely a critical change that permitted the deeper penetration of Atlantic moisture, an increase in the Moisture Flux Convergence (MFC) following the convergence of westerly winds inland (Fig. 2g–i, SI Fig. 6 and 10i), and ultimately the displacement of the maximum summer precipitation onto the forested area and its drier edge (Fig. 1a).

The shape of the MFC area seems to be defined by the position and intensity of the AEJ, indicating that in both control (CON) and afforestation (AFFO) simulations, the maximum MFC follows the southern margin of the AEJ (above about latitude 12°N; Fig. 2g,h). This is where the AEJ acts as barrier to the penetration of the monsoon westerlies, the Sahelian local source of moisture, and subdues the development of convergence^9,10^. Note that although the cooler SAT could promote subsidence, the weakening of the AEJ intensity increases the convergence at 10°N up to 600 mb (while there is weakening of the convergence north of the forest; Fig. 2i). The increase in MFC at 20°N is also associated with the increase in moisture advection northward (SI Fig. 6d–f).

A similar process of changes in the surface energy budget that lead to changes in the regional atmospheric circulation was observed in the North Australia afforestation simulations (SI Table 5b). The intensification of the wind patterns in the active environment of the monsoon^29^ led to a deeper penetration of the MFC over the afforested area in that region (SI Fig. 5,7 and 11i).

## Key vegetation elements influencing biosphere-atmosphere feedback

**Figure 3:**
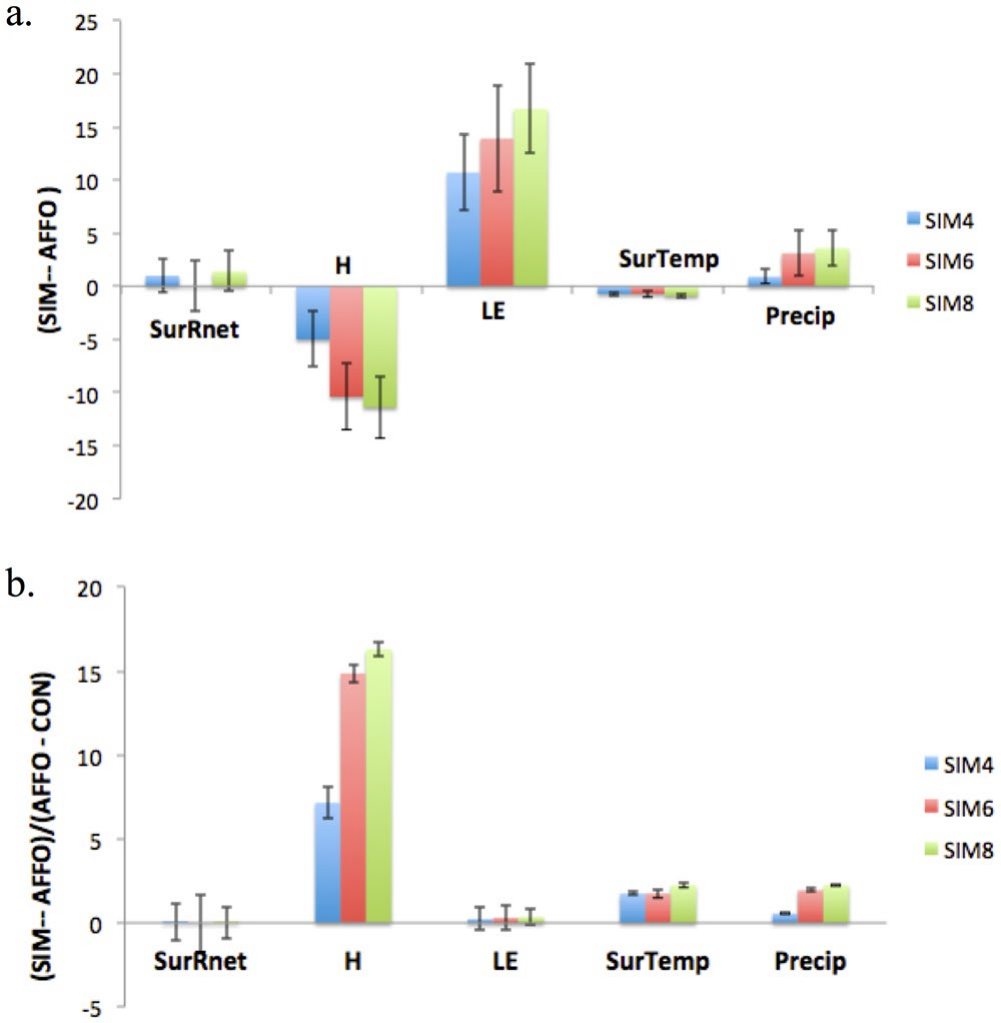
(**a**) Relative differences of SIM4 (albedo vegetation), SIM6 (vegetation height) and SIM8 (root-depth) from CON, compared to differences of AFFO from CON, in net radiation at the surface (SurRnet) [W m^−2^], sensible heat flux (H) [W m^−2^], latent heat flux (LE) [W m^−2^], surface air temperature (SurTemp) [°C] and precipitation (Precip) [mm day^−1^]. (**b**) The fraction of change for SIM – AFFO from the base deviation of AFFO - CON.

As noted above, the afforestation experiments were based on implementing a new plant functional type based only on the physical characteristics of a successful small-scale semi-arid afforestation system. We attempted to assess the hierarchy in the importance of the different physical characteristics, such as albedo, roughness (vegetation height), and root depth, in triggering the simulated changes in AEJ, SAT and precipitation (see Methods and SI Table S4). The results indicated that the dominant parameter in the afforestation experiment was most likely the increase in root depth, which allowed increased soil moisture mining and the initial transformation of the increased net radiation into enhanced evapotranspiration flux and surface cooling (Fig. 3a). Roughness (vegetation height, stand density) reinforced surface cooling by increasing surface-atmosphere aerodynamic conductivity. The proposed chain of events from increased root depth to the observed climatic changes in the large afforestation experiment are summarized in Fig. 5.

## Possible oceanic involvement in the Sahel and North Australian afforestation effects

**Figure 4:**
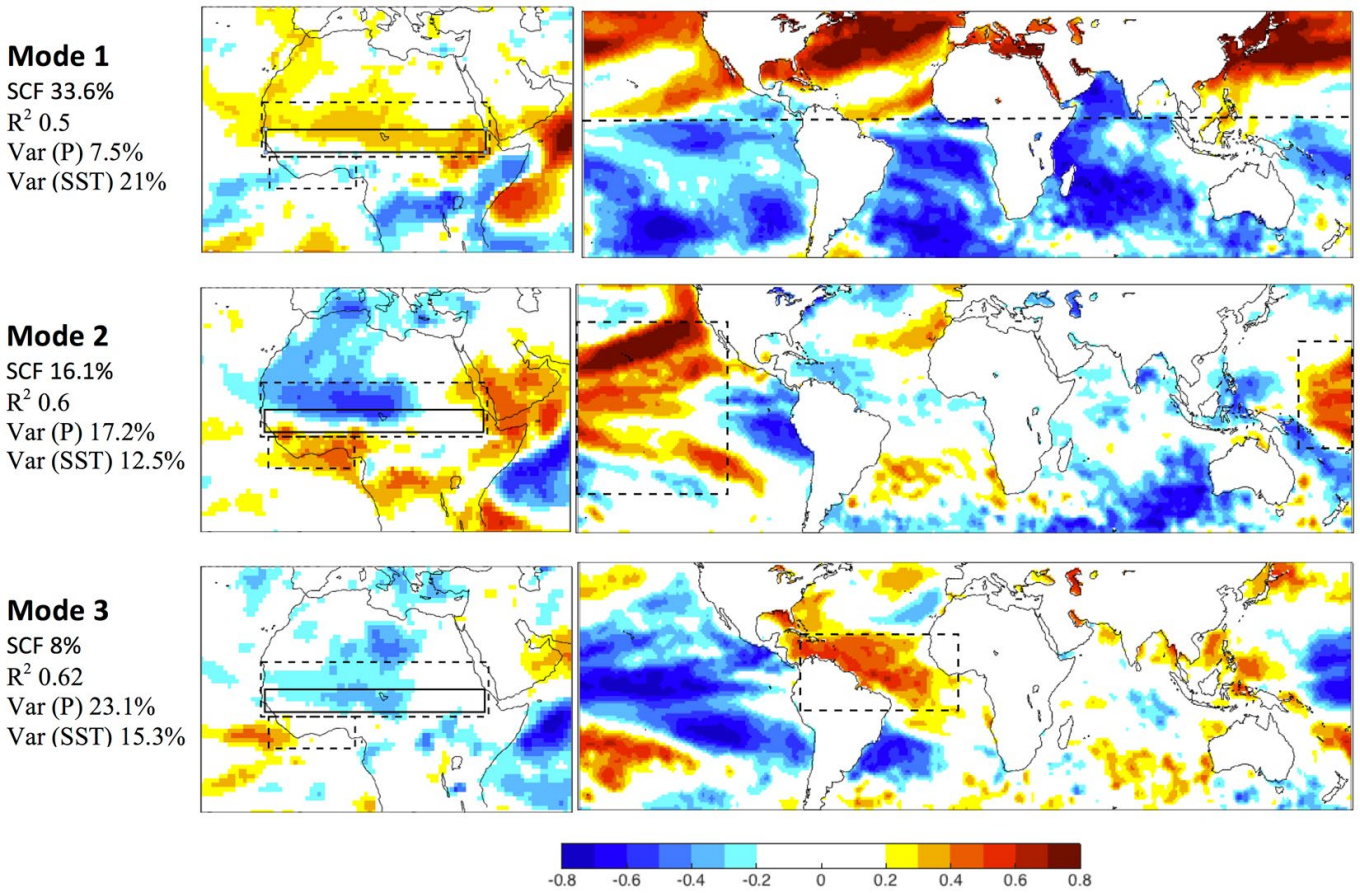
Leading mode derived from the MCA analysis of June to September (JJAS) precipitation (P) and sea surface temperature (SST) anomalies of AFFO-2d-S – CON-2d simulations from 1998 to 2012: P (left) and SST (right). In the P modes the dashed line represents the areas of the footprint (south Sahara) and the Gulf of Guinea coast; solid lines represent the afforested area (the Sahel). In the SST modes dashed lines represent the ocean basin area associated with the precipitation pattern over North Africa: Mode 1: Inter-hemispheric, Mode 2: Tropical pacific and Mode 3: Atlantic Ocean. The square covariance fraction (SCF) of each mode, the correlation (R^2^) between the MCA of the P and SST in the modes and the fraction of the variance (Var) of the given P and SST modes are presented. Maps were created using Matlab R2014b (http://www.mathworks.com/).

Although local land-atmosphere processes were proposed above as a plausible underlying mechanism for the afforestation effects, larger scale ocean and teleconnection effects can also be involved in enhancing, moderating or even driving the local effects. To check for such effects, we used a ‘maximum covariance analysis’ (MCA) procedure to derive a ‘statistical environment’ relating the precipitation over the Sahel and North Australia to sea surface temperatures (SSTs; see Methods). Over the Sahel the results showed a leading mode (33.6% of the covariance explained, Fig. 4, SI Fig. 8) that represents the increased precipitation over the afforestation footprint area in association with the northern hemisphere summertime, when solar insolation is increased over that domain (positive correlation) and decreases in the southern hemisphere (negative correlation). This creates the “inter-hemispheric gradient” mode noted by^7^. The second and the third leading modes (16% and 8% of covariance explained) show a reduction in Sahelian precipitation, either when the tropical Pacific is experiencing El Nino-Southern Oscillation (ENSO, characterized by its extreme phases: El Nino and La Nina) events^8,30^, amongothers and the warming of Atlantic Ocean or in relation to land-ocean temperature contrast^8,15,23^. Over North Australian all the first three leading modes (31.3%, 12.9% and 12.5% of the covariance explained, SI Fig. 9) represent increased precipitation north of the forested area inside the footprint area, with the first and second modes associated mainly with the ENSO and Indian dipole (IOD), where higher than average Indian Ocean SST near Australia enhance precipitation^31–33^. The second mode shows reduction of precipitation at the central of the footprint area and increased precipitation at the east west sides along the coastal areas. The third mode associated with increase SST over West and South Australia. The statistics of the leading MCA modes showed good correlation between the principal components in all the main patterns (leading modes). The results at the Sahel (North Australia) indicating high correlation 0.5 (0.6) and small variance 7.5% (8.9%) of the precipitation in the first mode strongly reinforces the persistence of increasing precipitation in the simulation results for the Sahel and North Australia domains during the monsoon (Fig. 1a, SI Fig. 2a). The overall conclusion of the MCA analysis is, therefore, that SST acts in concert with the simulated effects of the large-scale afforestation.

## Carbon sequestration potential

Finally, to assess the efficiency of a successful large-scale afforestation of semi-arid areas for climate change mitigation, the biogeochemical (BGC) effects due to the carbon sequestration potential should be estimated together with the direct biogeophysical (BGP) effects discussed above^2^. The relative importance of these two aspects can be assessed based on their radiative forcing, ${\rm{\Delta }}{R}{{F}}_{{C}{{O}}_{2}}$ vs. ${\rm{\Delta }}{R}{{F}}_{{energy}}$ (see ref.^1^). For a first approximation, our 15-year simulations indicate that despite the regional cooling the Sahel afforestation increased global net radiation at the top-of-atmosphere (TOA; note also the good agreement between the regional surface and TOA forcing reported in detail in SI Table 5) by **+**0.12 ± 0.9 W m^−2^ on the annual scale compared with the control, which is a net warming effect. We contrasted this with the results from the small-scale semi-arid afforestation system in Yatir as a generic indicator for the carbon sequestration potential under similar precipitation and radiation levels (annual 2.2 t C ha^−1^)^17^ and the current annual carbon uptake for the dry forest savanna of 0.12 t C ha^−1^ and 0.75 t C ha^−1^ in Sahel and north Australia, respectively^34^. This leads (see ref.^1,17^ for the calculation) to ${\rm{\Delta }}{R}{{F}}_{{C}{{O}}_{2}}$for the Sahel afforestation of 0.02 W m^−2^; it is a small cooling effect, but one which is cumulative over the lifetime of the forest. Therefore, for a first approximation, the small warming effects of the Sahel afforestation could be expected to balance in a period as short as ~6 years of carbon accumulation **(+**0.12 ± 0.9/0.02), which is insignificant in the lifetime of forests (>100 years)^35^. Once this balance is achieved (i.e. sum of BGP + BGC effects result in cooling), the positive feedbacks, including cooler surface, increased precipitation (which can sustain new forest on the extended footprint area), and the potential extension of the initial forest due to the larger moisture footprint, can support a large potential for carbon sequestration (and, in turn, wood and food production). This simplified perspective of the BGP vs. BGC effects assumes a carbon sequestration rate constant in time and with no effects of increasing concentrations of atmospheric CO_2_ and ignore other possible feedback with climatic conditions (consistent with observations in the 60 years old semi-arid forest in Yatir over the past ~20 years^36,37^).

Although the global semi-arid area is approximately 2000 × 10^6^ ha, using only the 200 × 10^6^ ha of the Sahel region and the annual net carbon uptake estimated to be above ~2-ton C ha^−1^, this translates into an annual carbon sequestration potential of ~0.2 Pg C. Assuming for a first approximation (and considering the scarcity of available data) that similar results applies to North Australia, the combined carbon sink of ca. 1/5 of the semi-arid regions could be ~0.4 Pg C annually. This is significant, considering that the estimated annual carbon sink of the entire terrestrial biosphere is approximately 2.5 Pg^38^, and it could provide a useful “wedge” in the global efforts to slow down the increase in atmospheric CO_2_ concentrations^39^.

**Figure 5:**
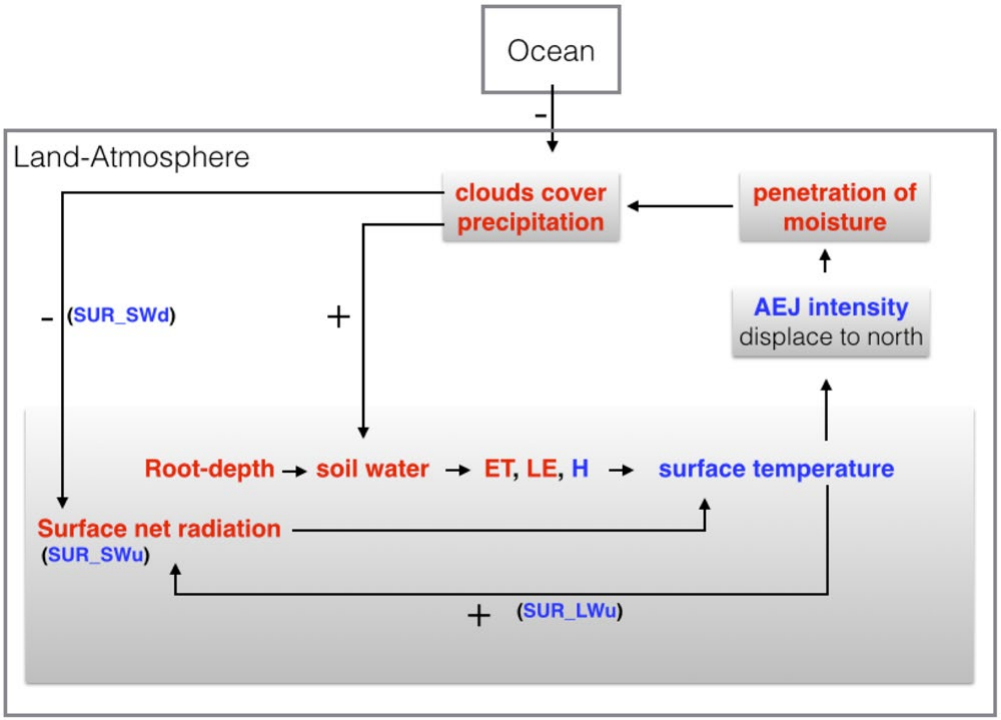
Schematic diagram for the sequence of processes underlying the increase in precipitation over the Sahel/N-Aust, as a consequence of land cover change from low-level vegetation to large-scale afforestation system. The starting point is the increased root depth associated with afforestation, and the associated increased in soil water mining. Red and blue colors indicate increase or decrease values, respectively.

As noted in the Introduction, previous studies (e.g.^2,11,19^) also reported model simulations that generally indicated increased precipitation associated with increasing vegetation. The present study, however, greatly extends earlier work with more comprehensive, higher resolution simulations, with a more realistic vegetation type, proposing new evolution of the effects and its amplification (Fig. 5), and considering Ocean teleconnections. Furthermore, this study indicates the potential benefits of afforestation of large semi-arid areas, which can be more efficient in terms of climate mitigation than afforestation in higher latitude regions, such as the Boreal region^1,26^. But it should also be noted that while this study highlight the benefits of forestation of hot semi-arid area under monsoon influence, there are semi-arid areas in cool and more continental regions that may involve different feedbacks. Clearly, further investigation is needed of afforestation potential in the vast semi-arid regions around the world, together with the possible climate feedbacks, the effects on water resources^40^ and carbon cycle dynamics.

## Methods

The model used is the Ocean-Land-Atmosphere-Model (OLAM)^41^, a new generation of GCM that allows numerical grid “telescoping” to increase spatial resolution using a flexible mesh refinement technique that operates on a hexagonal grid structure. Many of the physical parameterizations of OLAM (e.g. Turbulence, Cumulus convection, cloud microphysics, land surface) are adapted from the well-known and well-tested Regional Atmospheric Modeling System (RAMS)^42^ (see details in SI Table 1).

For the following experiments, we used OLAM coupled with the Rapid Radiative Transfer Model Global version (RRTMG)^43^ for a lower grid resolution (characteristic length scale 200 km). The model was assigned a land cover classification (PFT; see details in SI Fig. 1 and Table 2) and monthly NDVI for the year 2003 (the middle year and the period for the high resolution simulations), based on MODIS products with a spatial resolution of 5-km and 1-km, respectively^44^. The land cover classification remained constant throughout the simulation.

We set up two types of simulations: the control (CON) and the afforestation (AFFO). The first had the current state of PFT over the area of interest, and the afforested scenario used a land cover represented by a new PFT of semi-arid planted forest based on the physical characteristics of the semi-arid Yatir forest. The new PFT was implemented uniformly over the entire area, completely replacing the existing PFT over the Sahel [16°W-40°E, 10–15°N; 2.6 10^6^ km^2^] and North Australia [122°-146° E, 17°-22°S; 2.1 10^6^ km^2^] in a land-area band with a minimum annual precipitation of 300 mm (based on the of Yatir afforestation system assumed to be at the “dry timberline forest”)^17^. The values of the parameters characterizing the Yatir forest were based on an inventory of a field-base study starting in 2000 (see details in SI Table 2).

The main experiments were based on nearly 17-year simulations (April 1996 to December 2012), with a spin-up period, for these and other simulations, that was always at least 1.5 years based on previous experience^45,46^ and our own observations. The simulations were performed with a unified “Characteristic Length Scale” (CLS, defined as the square root of the area represented by a grid element) of 200-km (~2 degree), with control (CON-2d) and afforestation over the Sahel (AFFO-2d-S) and North Australia (AFFO-2d-NA) (see details in Table 1 for list of simulations). To evaluate the effects of model resolution on the simulation^46^, we also carried out simulations with a 200-km CLS globally, but 50-km CLS over Africa and the Indo-Australian Ocean domains (see SI Fig. 1). The simulated period in this case was January 2000 to December 2005, but the analysis reported here is for summer months (Jul-Sept, JAS for Sahel and Jan-Mar, JFM for Australia) during 2003–2005. Note that these shorter (~6 y) simulations, being consistent with the more robust 15-y simulations, only aimed to confirm that resolution was not a major factor underlying the results.

**Table 1:** Summary of the numerical experiments.

Experiments	Boundary condition	Period
CON-2d	Atmospheric grid structure of CLS 200 km unified globally.	17 years
	Monthly climatological SST and sea ice averaged over the period 4/1996 to 12/2012, using NCEP/NCAR reanalysis.	
AFFO-2d-S	Atmospheric grid structure of CLS 200 km unified globally.	17 years
	Forced by Yatir PFT over the Sahel [16°E-40°W, 10–15°N], monthly climatological SST and sea ice averaged over the period 4/1996 to 12/2012, using NCEP/NCAR reanalysis.	
AFFO-2d-NA	Atmospheric grid structure of CLS 200 km unified globally.	17 years
	Forced by Yatir PFT over Australia [115–150°E, 17–22°S], monthly climatological SST and sea ice averaged over the period 4/1996 to 12/2012, using NCEP/NCAR reanalysis.	
CON-0.5d-S	Atmospheric grid structure of CLS 200 km unified globally	6 years
	and gradually increased to CLS 50 km over Africa. Monthly climatological SST and sea ice averaged over the period 1/2000 to 12/2005, using NCEP/NCAR reanalysis.	
CON-0.5d-NA	Atmospheric grid structure of CLS 200 km unified globally	6 years
	and gradually increased to CLS 50 km over Australia. Monthly climatological SST and sea ice averaged over the period 1/2000 to 12/2005, using NCEP/NCAR reanalysis.	
AFFO-0.5d-S	Atmospheric grid structure of CLS 200 km unified globally	6 years
	and gradually increased to CLS 50 km over Africa. Forced by Yatir PFT over the Sahel [16°E-40°W, 10–15°N], monthly climatological SST and sea ice averaged over the period 1/2000 to 12/2005, using NCEP/NCAR reanalysis.	
AFFO-0.5d-NA	Atmospheric grid structure of CLS 200 km unified globally	6 years
	and gradually increased to CLS 50 km over Australia. Forced by Yatir PFT over North Australia [115–150°E, 17–22°S], monthly climatological SST and sea ice averaged over the period 1/2000 to 12/2005, using NCEP/NCAR reanalysis.	

Seven numerical experiments were carried out in this study using OLAM and are summarized in Table 1. Control experiments (CON_2d_, CON_0.5-S_ and CON_0.5d- NA_) were integrated from the initial condition taken from at least 1.5 years spin-up experiment for the lower and higher grid resolution. The simulations were forced by the current state of the vegetation classification. The large-scale afforestation experiments (AFFO_2d-S_, AFFO_2d- NA,_ AFFO_0.5d-S_, AFFO_0.5d- NA,_) implemented with a land cover classification representing a new PFT of semi-arid planted forest with physical characteristics based on the Yatir semi-arid site (see Table S2). Abbreviations: control simulation (CON), afforestation scenario (AFFO), Sahel (S), North Australia (NA), two and a half degree grid horizontal resolution (2d and 0.5d), characteristic length scale (CLS), sea surface temperature (SST), plant function type (PFT).

All simulations were carried out with a vertical structure of 50 layers, ranging in thickness from 50 m near the surface to 2000 m at high altitudes. The soil model extends to a depth of 5 m and is discretized into 21 layers. Vertical water transport between soil layers is represented by Richards equation and includes a flux at the lower boundary that is driven by hydraulic head in the lowest soil layer and a boundary head value that applies at a hypothetical layer beneath it. The lower boundary head is computed based in part on the depth of the water table, which in the Sahel region is usually too deep to provide a moisture source to the soil layers. Therefore, in the present simulations, saturated soil would drain through the lower boundary, while dry soil will lose little to no water through the lower boundary because of the low hydraulic conductivity in dry soils. The time step of the model was set for 45 s for the dynamical processes and 1800 s for the radiation calculation. Analysis of the response to large-scale afforestation was estimated from the mean difference between the CON and AFFO experiments. The seasonal values for summer time in Africa (JAS) and in Australia (JFM) are produced by averaging the corresponding monthly mean values. The SST and Sea-Ice values were prescribed with NCEP/NCAR Reanalysis v2^47^ for each month of the period.

To investigate in more detail some specific factors, we used the additional analyses as follows: first, the simulations for 15 years (1998 to 2012) with a 200-km CLS used to assess the links between the Easterly Jets (AEJ and AUSEJ) and precipitation in the context of large scale circulation, we calculated the Moisture Flux Convergence (MFC) term as done by others^48^. Second, to assess the importance of plant functional type (PFT) characteristics on generating the initial condition for climate change over the Sahel, a series of short-term simulations were carried out (summarized in SI Table 4), in which the physical characteristics of the Yatir PFT was modified from that of bare soil to that of dense tree forest. The data analyzed were for one year for summer time (JAS), following the 1.5 year spin-up (based on consistency with the long simulations and our experience that the signal obtained is strong enough to emphasize the sensitivity, or lack of it, of the simulated system). The CLS was four degrees (~400 km) and 50 vertical atmospheric levels. The SST and sea-ice were prescribed using the NCEP reanalysis for the relevant years. To assess the ocean influence on the precipitation over the Sahel and North Australia afforestation area, we employed the maximum covariance analysis (MCA). The MCA is well discussed in Wallace^49^ and Bretherton^50^, and it can be considered as a generalization of principal component analysis (PCA), and indeed reduces to it when two fields are identical. The MCA is usually applied to two data fields, in order to identify the pairs of coupled spatial patterns that explain the largest covariance between the two variables. The MCA procedure was applied for 15 years (1998 to 2012) with a 200-km CLS in June to-September (JJAS) and December to March (DJFM) in Sahel and North Australia, respectively for the mean bias (AFFO minus CON) of SST and precipitation. Precipitation was calculated for the area of North Africa [12°W-42°E, 32°S-62°N] and North Australia [122°-146° E, 12°-22°S] and the prescribed SST was prepared for a global scale (50°S, 50°N). The following statistics are presented: (1) the correlation coefficient (R^2^) between the MCA of the precipitation and SST of each mode (see Fig. 4); (2) the fraction of covariance explained by each mode is given by the percentage format of the square covariance fraction (SCF), $SCF(i)={L}_{i}^{2}/{\sum }_{j=1}^{q}{L}_{j}^{2}\ast 100$, were *L* is a singular value of the cross-covariance matrix, and *q* is the number of the significant Eigen values; and (3) the fraction of the variance of a given precipitation *Var*(*Pi*), explained by the ratio of the variance of the appropriate principal component to the total variance of the vector, which was obtained by summing all the principal components, $Var({P}_{i})=var(P{C}_{P(i)})/{\sum }_{j=1}^{q}var(P{C}_{P(j)})$.

### Uncertainty of the OLAM simulations

A series of model runs were carried out to evaluate the ability of OLAM to simulate global radiation and climatological patterns. OLAM’s radiation scheme was evaluated against the database of NOAA’s Cloud and the Earth Radiation Energy System (CERES) on a 1 × 1° grid. For the climatological evaluation, OLAM surface temperature field was compared to the NCEP/NCAR reanalysis^47^ 2.5-degree and to the Climatic Research Unit (CRU) TS3.21^51^ (1901–2013) on a 0.5 × 0.5° grid. The precipitation field was compared to the Global Precipitation Climatology Project (GPCP) v2.2^52^ (1979–2013) on a 2.5 × 2.5° grid, the CRU TS3.21^51^ (1901–2013) on a 0.5 × 0.5° grid, and the Tropical Rainfall Measuring Mission Project (TRMM), Product 3B43, v7^53^, (2000–2013) on a 0.25 × 0.25°grid. The comparisons for 2.5-degree grid resolution were performed for the monthly average of the years 1998–2012. The spatial patterns of the climatology in the observations and in the CON-2d, CON-0.5d-S, and CON-0.5d-NA model experiments indicated that the main features of the large-scale circulation and precipitation in the Sahel and North Australia were reasonably well reproduced in comparison to observations and reanalysis. The simulations with higher resolution indicate over estimation in precipitation and SAT compare to underestimation in the simulations with lower resolution (SI Fig. 3 and SI Fig. 4). For the model experiment CON-2d, in Africa, the position of the Saharan heat low (SHL) and its strength also compare well with the observations (SI Fig. 12a,b). Associated with the SHL are strong westerly monsoon (WM) winds around ~10°N to the south of the heat low and northeasterly winds to the north in observations. The model simulates the northeasterly winds to the north of the heat low well, but somewhat underestimates the core of WM by 2 ms^−1^, mainly to the south (SI Fig. 10g,h). As a result, the model-simulated monthly mean precipitation does not extend as far northwards as the data indicate, with the mean Sahel rainfall being underestimated by less than 0.2 ± 1.1 mm d^−1^ and 0.4 ± 1.0 mm d^−1^ relative to CRU and GPCP respectively (Fig. 1b and SI Fig. 10a–c). The underestimation of the Sahel rainfall in the model is associated with a cold bias of 2.8 ± 0.8 °C and 1.5 ± 0.8 °C in SAT relative to CRU and NCEP respectively (Fig. 1e and SI Fig. 10d–f) and a relatively weak meridional temperature gradient over North Africa, which is linked to the weak vertical shear of zonal wind, AEJ (underestimate the core by ~2 ms^−1^), and the weak surface westerly winds over the Sahel (SI Fig. 10g,h, 12). Underestimation of the MW and AEJ’s strength is consistent with previous modeling studies, with a range of ±3 ms^−1^ ^9,54–56^. In Australia, the model simulated the main regional circulation patterns: a high-pressure system developing at 30°S, 120–135°E and the jet maximum located at approximately 17°S. The position of the key features of the region (SI Fig. 11g,h, 13) are the AUSEJ core at 850 mb and over estimate by 2 ms^−1^, the Indonesian-Australian Summer Monsoon (I-ASM) core at 900 mb and under estimate by up to 6 ms^−1^, and the westerly winds Sub Tropical Jet (STJ) at 200 mb. All the features’ locations are reproduced reasonably well. The rainfall being underestimated by 0.3 ± 2.1 mm d^−1^ and 0.5 ± 2.0 mm d^−1^ relative to CRU and GPCP respectively (SI Fig. 11a–c). The SAT cooler by 2.0 ± 1.6 °C, 2.7 ± 1.8 °C relative to CRU and NCEP respectively (SI Fig. 11e,f) (SI Fig. 11e,f). The long-term variability and spatial features, such as SAT and precipitation for both Sahel and North Australia (Fig. 1c,f and SI Fig. 2c,f) are also reproduced reasonably well in comparison with observations and reanalysis. The minimum bias, which is in the range of the uncertainty of the other modeling simulations in the region, for SAT and precipitation is observed in JAS and JFM, respectively^57^.

With respect to model observations bias, note that there are also biases between the different ‘observation’ databases: Within the Sahel the precipitation in GPCP is overestimated by 0.2 ± 0.4 mm d^−1^ relative to CRU and the temperature in NCEP is cooler by 1.3 ± 0.4 °C relative to CRU; in North Australia, the precipitation in GPCP is overestimated by 0.2 ± 0.5 mm d^−1^ relative to CRU and CRU is cooler by 0.8 ± 0.5 °C with respect to NCEP.

## Electronic supplementary material

Supplementary information
