# Supplementary material for: Large-scale semi-arid afforestation can enhance precipitation and carbon sequestration potential
Source: Sci Rep. 2018 Jan 17;8:996. doi: 10.1038/s41598-018-19265-6 (PMC5772497; doi:10.1038/s41598-018-19265-6)
Supplement: Supplementary file 1 — Supplementary information [file 41598_2018_19265_MOESM1_ESM.pdf]

Supplementary Information

Large-scale semi-arid afforestation can enhance precipitation and carbon sequestration potential

Gil Yosef[1], Robert Walko[2], Roni Avisar[2], Fedor Tatarinov[1], Eyal Rotenberg[1] and Dan Yakir[1]*

[1]Earth and Planetary Sciences, Weizmann Institute of Science, Rehovot, Israel

[2]Rosenstiel School of Marine and Atmospheric Science, University of Miami, Miami, Florida

The model used is the Ocean-Land-Atmosphere Model (OLAM) *(Walko and Avissar, a.2008, b.2008; Walko et al., 2000)*, which is a relatively new global General Circulation Model (GCM) that allows "nesting down" to any desired resolution using a flexible mesh refinement technique that works on a hexagonal grid structure. The physical climate components of OLAM are based on a global version of the well-known Regional Atmospheric Modeling System (RAMS) (see Table S1).

Table S1. Model components for the simulations carried out during this research

Model aspects	Chosen scheme
Dynamics	Non-hydrostatic,<br>height-vertical coordinate
Grid structure	Arkawa-C grid stage<br>Hexagon grid cells, cut-cell method over terrain
Radiative transfer	RRTMg and Harrington scheme for simulation with 200 km and 50 km grid resolution respectively
Turbulence	Smagrorinsky (1963) deformation-K closure scheme with stability modifications by Lilly (1962) and Hill (1974)
Cumulus convection	Modified KF_eta
Cloud microphysics	Single-moment bulk scheme - Walko et al. (1995), with look -up table bin-micro for autoconversion and sedimentation (Feingold et al., 1998) and non-hydrometeor thermo (Walko et al., 2000)
Land surface	Soil/vegetation/snow parameterization - LEAF 4
Ocean surface	Monthly sea surface temperature, sea ice cover (NCEP)

Table S2. Biophysical parameters for various vegetation classes and the new suggested vegetation class of Yatir.

Parameterization	Bare soil/desert	Short grass	Yatir	Evergreen needleleaf	Evergreen broadleaf
Albedo veg green	0	0.21	0.12	0.14	0.14
Albedo veg brown	0	0.43	0.24	0.24	0.24
Emissivity	0	0.96	0.97	0.97	0.97
Simple ratio NDVI	0	5.1	5	5.4	4.1
Total area index	0	4	3 max	8	7
Steam area	0	1	0.1	1	1
Veg clump	0	0	1	1	1
Veg frac cover	0	0.75	0.56	0.8	0.9
Veg height [m]	0	0.3	10	20	32
Root depth	0	0.7	1.5	5	5
Dead frac	0	0.7	0	0	0
Min canopy resistance	0	100	286	500	500

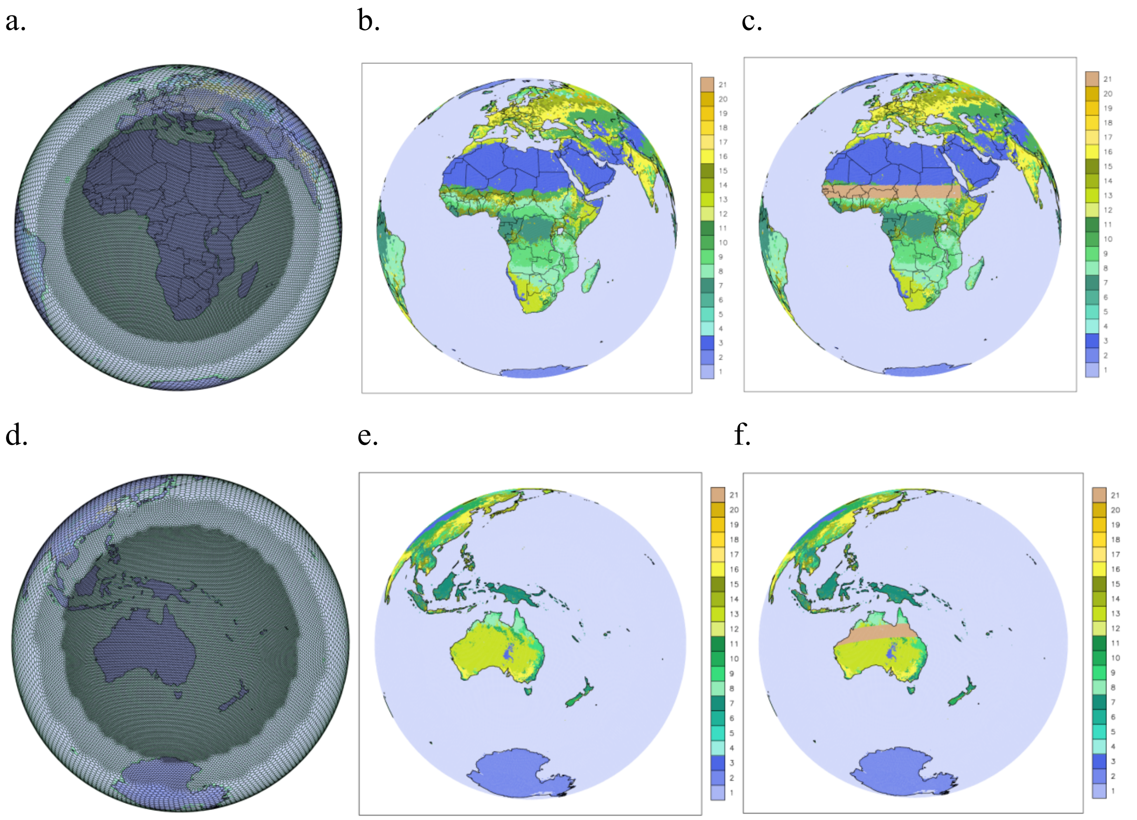

Figure S1. (a) OLAM grid structure: variable resolution, globally 200 km grid size and gradual increase to 50 km. (b) LEAF land cover class's type for the CON simulation and (c) AFFO simulation with Yatir vegetation type (number 21) over the Sahel. The same grid structure and land cover obtain for North Australia (d) to (f). Maps in this figure were created using NCL (https://www.ncl.ucar.edu/).

Table S3. LEAF land cover class's type

Index	Land cover type
0	Ocean
1	Lakes, rivers, streams
2	Ice cap/glacier
3	Desert, bare soil
4	Evergreen needleleaf tree
5	Deciduous needleleaf tree
6	Deciduous broadleaf tree
7	Evergreen broadleaf tree
8	Short grass
9	Tall grass
10	Semi-desert
11	Tundra
12	Evergreen shrub
13	Deciduous shrub
14	Mixed woodland
15	Crop/mixed farming, C3 grassland
16	Irrigated crop
17	Bog or marsh
18	Wooded grassland
19	Urban and built up
20	Wetland evergreen broadleaf tree
21	YATIR Evergreen needleleaf tree

To address the question of vegetation cover influence on triggering positive mechanism, eight simulations were carried out during the period of 2000-2002: (1) as the control simulation SIM1 $_{(CON)}$, which represents the state condition of the Sahel, with mixed vegetation of semi-desert, short grass and tall grass, (2) simulation with afforestation scenario SIM2 $_{(AFFO)}$ over the Sahel region. Forest parameters were taken from semi-arid forest parametrization (Yatir forest, Northern Negev, Israel) (Table S2). Other simulations (SIM3 to SIM8) had the same vegetation parametrization as the SIM2 $_{(AFFO)}$ with one parameter in each simulation replaced by the corresponding value from short-grass (sh) or evergreen broadleaf (ev) parameterizations, which represent the two end-members of the vegetation spectrum. The changed parameters were: a) vegetation albedo (albv), b) vegetation height (h) and c) root depth (rd) (Table S4). Then the biases values for SIM3 to SIM8 were compared to the biases for SIM2 $_{(AFFO)}$-SIM1 $_{(CON)}$ bias. The following output variables were analyzed for July-August-September (JAS): surface and top of atmosphere energy budget, surface temperature and precipitation over the Sahel (solid line) [16^0 E-40^0 W, 10^0-15^0 N] and for precipitation also the footprint area were calculated (dash line) [16^0 E-40^0 W, 10^0-20^0 N] (Fig. S3).

Table S4. Simulations of the biophysical parameters for Yatir type forest and the adjustment values (h – canopy height, rd-root depth and albv –albedo vegetation) for the Short grass (sh) and Evergreen Broadleaf (ev) vegetation simulations.

Simulations	Parameterization	Short Grass	Evergreen Broadleaf
Triggering positive mechanism			
SIM3 (albv-sh)	Albedo veg green	0.21	-
	Albedo veg brown	0.43	-
SIM4 (albv-ev)	Albedo veg green	-	0.17
	Albedo veg brown	-	0.21
SIM5 (h-sh)	Veg height [m]	0.3	-
SIM6 (h-ev)	Veg height [m]	-	32
SIM7 (rd-sh)	Root depth [m]	0.7	-
SIM8 (rd-ev)	Root depth [m]	-	5
Impact of regional-scale land cover change			
SIM9 (d)	PFT Bare-soil/desert (albedo 0.41)		
SIM10 (sh)	PFT Short grass		
SIM11 (br)	PFT Evergreen Broadleaf		

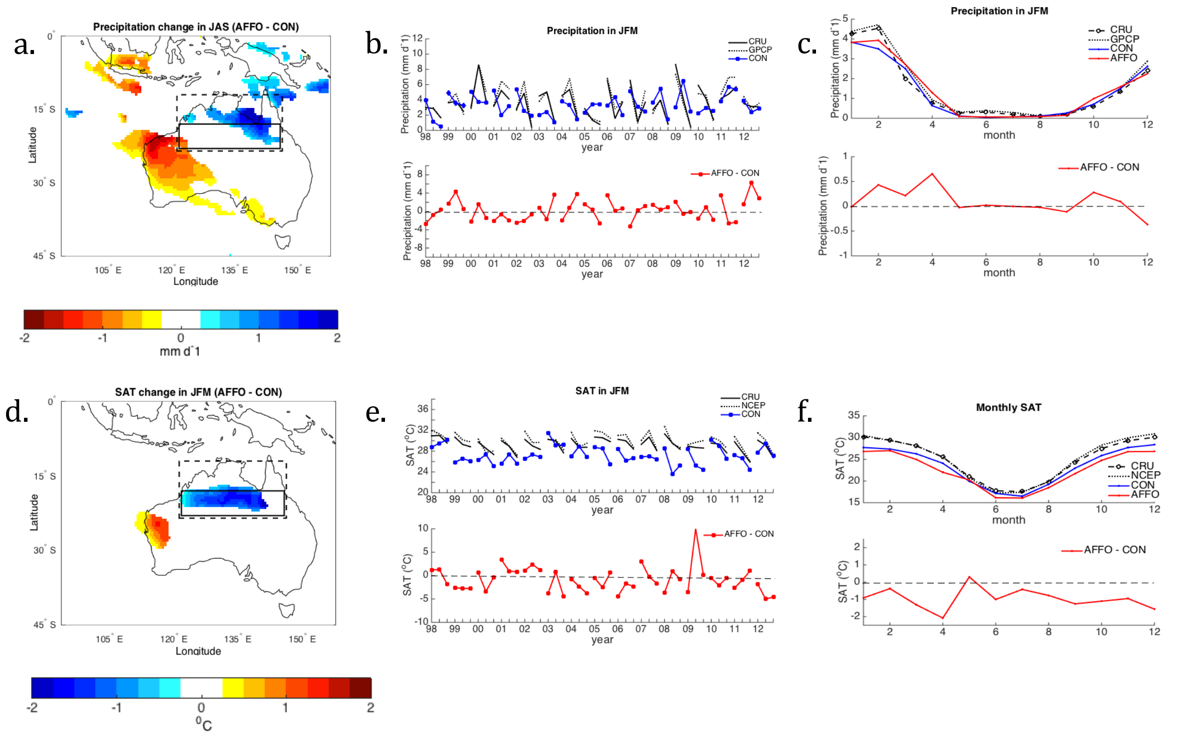

Figure S2. Observations (CRU, GPCP and NCEP) and model-simulated precipitation and surface-air-temperature (SAT) over Africa between the years 1998 and 2012. Precipitation: (a) Spatial patterns of observed seasonal mean of January-February-March (JFM) bias between afforestation (AFFO-2d-NA) minus control (CON-2d) with 95% confidence level, (b) Time series of rainfall over the afforested area for consecutive JFM and (c) Seasonal evolution over the afforested area. The same for (d-e) SAT. The solid and dashed lines cover the afforested area [17^0-22^0 S, 122^0 -146^0 E] and the footprint area respectively [12^0-22^0 S, 122^0 -146^0 E]. Maps in this figure were created using Matlab R2014b (http://www.mathworks.com/).

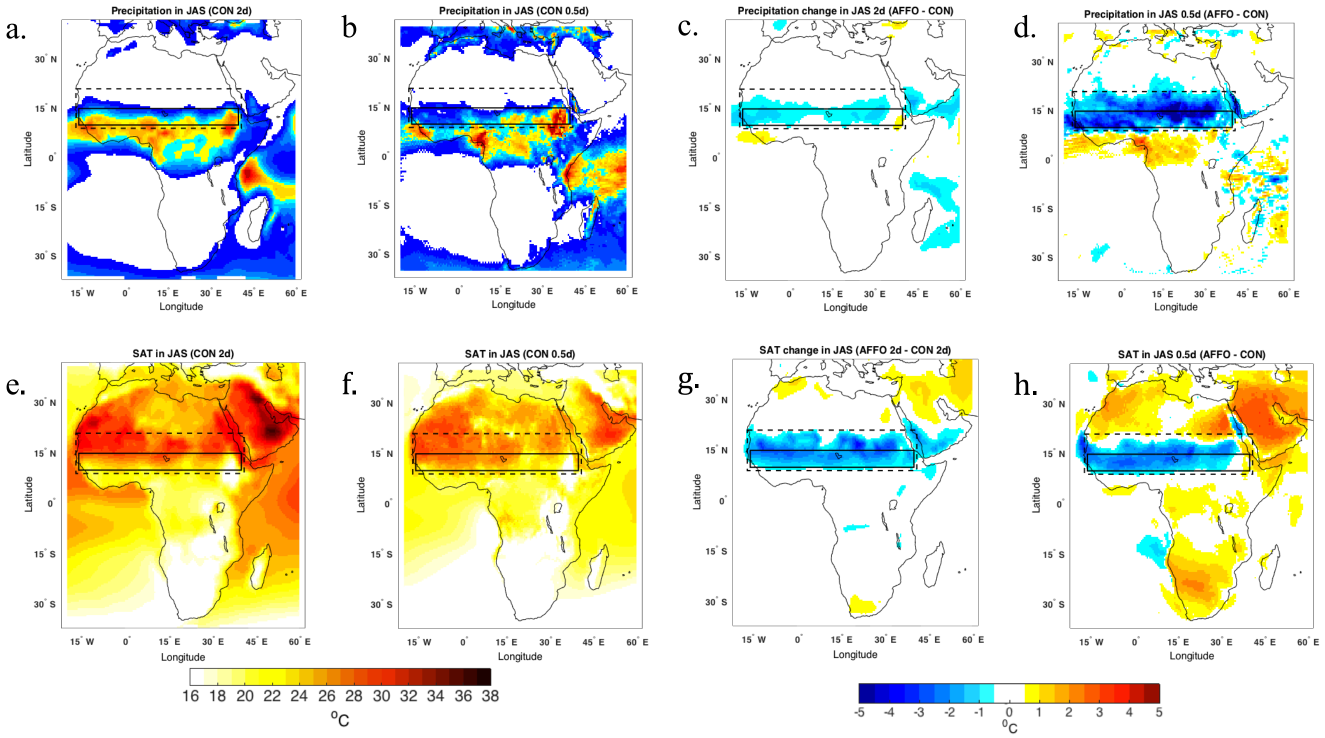

Figure S3. Model simulations of Precipitation and SAT in JAS during the years 2003-2005 over Sahel. Precipitation: (a) CON-2d, (b) CON-0.5d-S, (c) AFFO-2d-S – CON-2d and (d) AFFO-0.5d-S – CON-0.5d-S. SAT: (e)-(h) the same as (a)-(d). The afforested area indicated in the solid line [10^0-20^0 N, 16^0 W-40^0 E], dash lines cover the footprint area [10^0-20^0 N, 16^0 W-40^0 E]. Maps in this figure were created using Matlab R2014b (http://www.mathworks.com/).

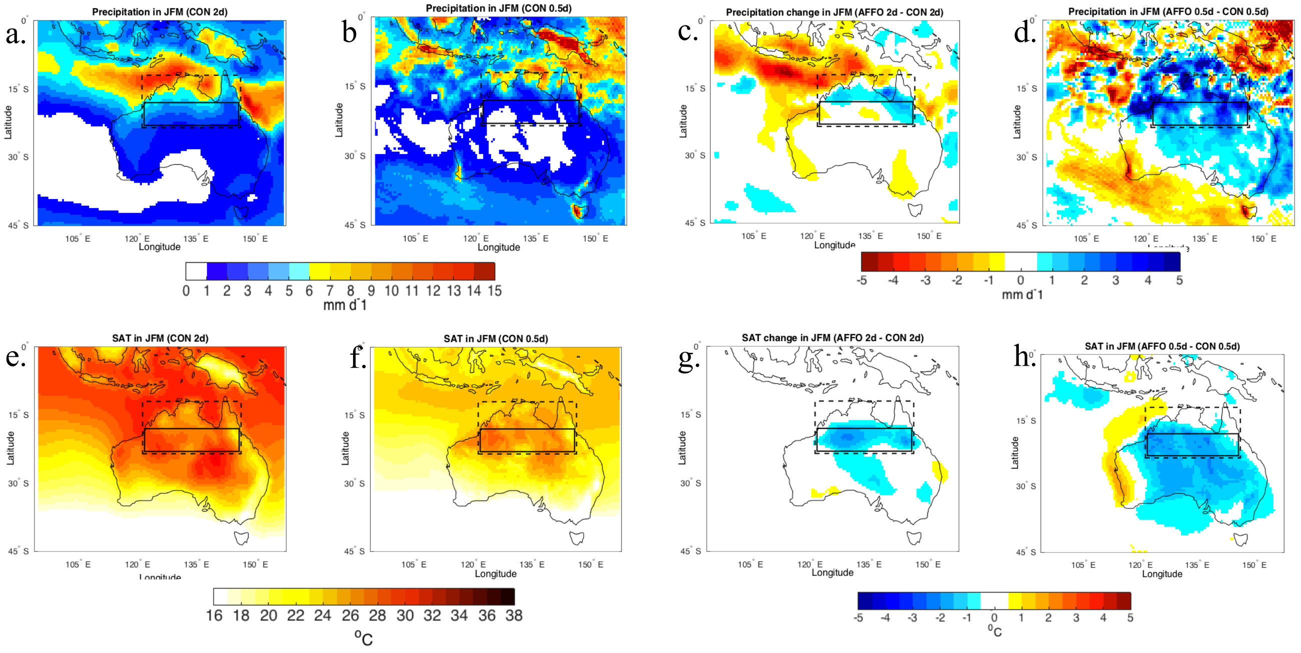

Figure S4. Model simulation of Precipitation and SAT in JFM during the years 2003-2005 over N-Aust. Precipitation: (a) CON-2d, (b) CON-0.5d-NA, (c) AFFO-2d-NA – CON-2d and (d) AFFO-0.5d-NA – CON-0.5d-NA. SAT: (e)-(h) the same as (a)-(d). The afforested area indicated in the solid line [17^0-22^0 S, 122^0 -146^0 E], dash lines cover the footprint area [12^0-22^0 S, 122^0 -146^0 E]. Maps in this figure were created using Matlab R2014b (http://www.mathworks.com/).

Table S5. Sahel (a.) and N-Aust (b.) energy budget of the CON and AFFO scenarios. Surface (SUR) and Top of Atmosphere (TOA) energy budget components during the period 1998-2012 over a. Sahel [16^0 E-40^0 W, 10^0-15^0 N], in JAS, and b. N-Aust [17^0-22^0 S, 122^0 -146^0 E], in JFM. The experiments CON-2d, AFFO-2d-S and AFFO-2d-NA: radiative components: Up (u) and Down (d), Short and Long wave (SW and LW), and total net radiation (Rnet), Surface non-radiative components: sensible (H) and latent (LE) heat flux, Evapotranspiration (ET) and Surface Air Temperature (SAT). Cloud forcing also calculate with subtracting the full sky from the clear sky. Spatial standard deviation (std) was calculated for the given region. Units: Energy components [W m-2], ET [mm day^{-1}] and SAT with [^{0}C].

a.

	CON				AFFO				AFFO-CON				cloud forcing
	full sky	std	clear sky	std	full sky	std	clear sky	std	full sky	std	clear sky	std	
SUR-SWd	289.4	11.6	324.6	2.3	274.2	11.3	322.3	2.2	-15.2	1.9	-2.3	0.2	-13.0
SUR-SWu	78.5	9.9	86.3	9.8	63.7	9.6	71.8	9.6	-14.8	1.1	-14.5	1.2	-0.3
SUR-LWd	358.9	12.4	352.2	10.6	361.3	12.3	352.5	10.4	2.4	0.8	0.3	0.6	2.1
SUR-LWu	454.2	14.7	454.1	14.7	446.3	14.5	446.1	14.5	-8.0	0.9	-8.0	0.9	0.0
SUR-Rnet	115.6	4.5	136.5	9.7	125.5	4.4	156.9	9.1	9.9	1.2	20.5	1.4	-10.5
TOA-SWd	413.9	0.0	413.9	0.0	413.9	0.0	413.9	0.0	0.0	0.0	0.0	0.0	0.0
TOA-SWu	126.5	6.3	100.2	8.7	125.6	6.4	87.4	8.4	-0.9	1.5	-12.8	1.0	11.9
TOA-LWu	272.3	7.6	291.0	8.6	263.6	7.4	287.2	8.3	-8.6	1.0	-3.8	0.4	-4.9
TOA-Rnet	15.1	5.9	22.7	8.5	24.6	5.9	39.3	7.9	9.5	1.4	16.6	1.2	-7.1
H	55.3	5.1			49.7	5.1			-5.6	0.9			
LE	64.7	13.9			81.6	13.7			16.9	2.5			
ET (mm/day)	2.2	0.5			2.8	0.5			0.6	0.1			
SAT [C]	24.7	0.4			23.4	0.4			-1.3	0.14			

b.

	CON				AFFO				AFFO-CON				cloud forcing
	full sky	std	clear sky	std	full sky	std	clear sky	std	full sky	std	clear sky	std	
SUR-SWd	261.1	12.0	297.3	2.3	257.8	11.3	297.0	2.2	-3.4	2.3	-0.3	0.1	-3.0
SUR-SWu	54.4	10.0	61.0	9.9	53.2	10.0	59.6	9.9	-1.2	0.5	-1.4	0.6	0.2
SUR-LWd	355.0	12.4	346.4	10.6	354.8	12.4	345.5	10.7	-0.2	0.6	-0.9	0.4	0.7
SUR-LWu	443.2	14.5	443.1	14.5	438.3	14.5	438.2	14.5	-4.9	0.5	-4.9	0.5	0.0
SUR-Rnet	118.5	4.7	139.7	9.8	121.1	4.8	144.8	9.6	2.6	1.3	5.1	0.6	-2.5
TOA-SWd	385.0	0.0	385.0	0.0	385.0	0.0	385.0	0.0	0.0	0.0	0.0	0.0	0.0
TOA-SWu	105.1	6.7	77.3	8.7	106.8	6.5	76.1	8.7	1.7	2.0	-1.2	0.5	3.0
TOA-LWu	274.4	7.6	285.6	8.4	271.5	7.5	283.9	8.4	-2.9	0.9	-1.7	0.2	-1.1
TOA-Rnet	5.5	5.9	22.0	8.4	6.7	6.2	25.0	8.4	1.1	1.6	3.0	0.5	-1.8
H	64.0	5.2			57.9	5.1			-6.1	0.8			
LE	66.2	13.9			74.9	13.7			8.7	1.9			
ET (mm/day)	2.3	0.5			2.6	0.5			0.3	0.1			
SAT [C]	27.1	0.5			26.2	0.5			-0.9	0.1			

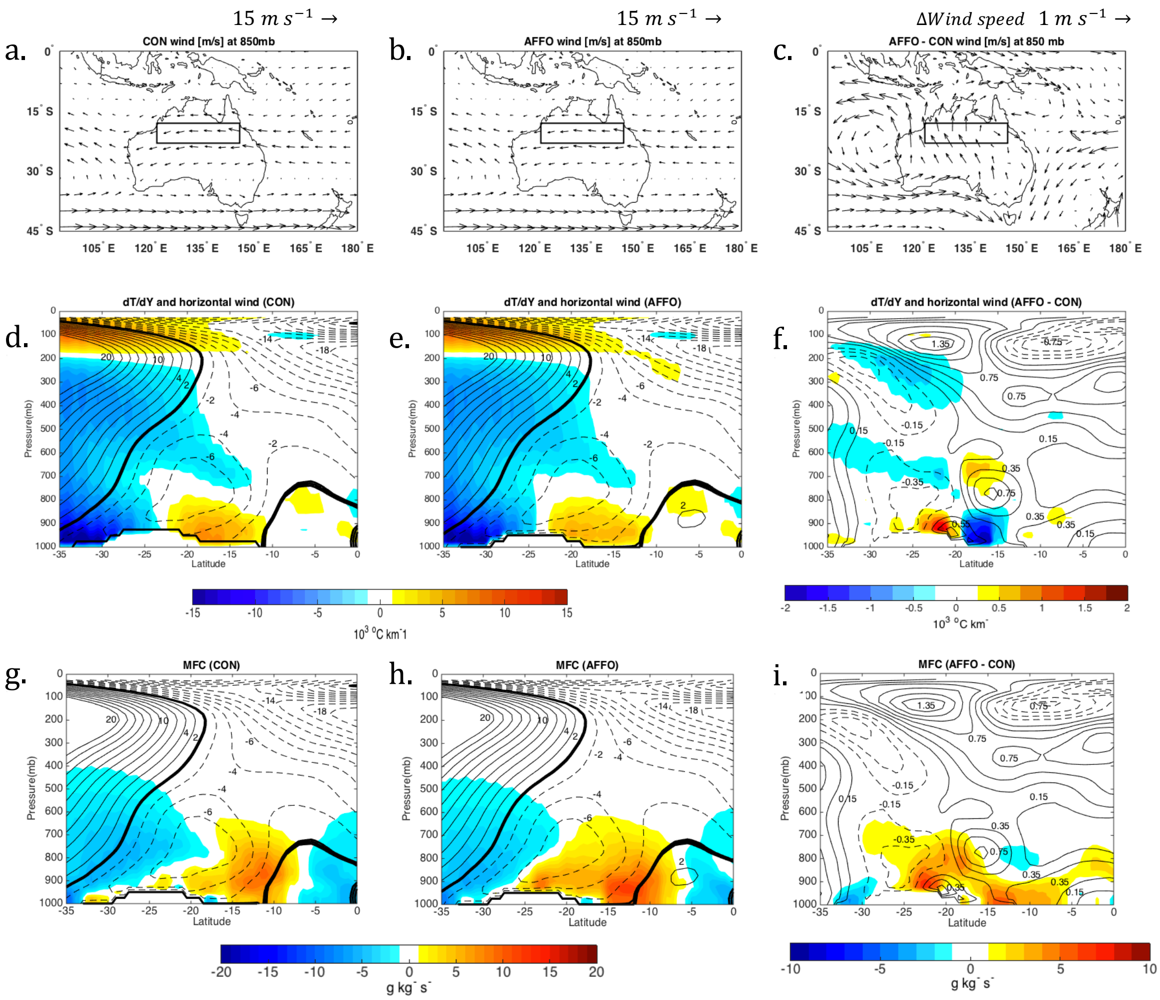

Figure S5. Model simulated seasonal mean of January-February-March (JFM) during the period 1998 -2012 over Africa, of zonally (130^0-135^0E) averaged: (a)-(c) spatial patterns of wind at 850 mb, (a) control (CON-2d), (b) afforestation (AFFO-2d-NA) and (c) bias between afforestation (AFFO-2d-NA) minus control (CON-2d). The same analysis for: (d)-(f) meridional temperature gradient dT/dy [10^3 ^{0}C km^{-1}], (g)-(i) the total Moisture Flux Convergence (MFC) convergence/divergence (positive/negative) with zonal wind [m s^{-1}]. Solid lines and dash line represents easterly and westerly winds intensity respectively. Units in [10^3 g kg^{-1} s^{-1}]. Maps in this figure were created using Matlab R2014b (http://www.mathworks.com/).

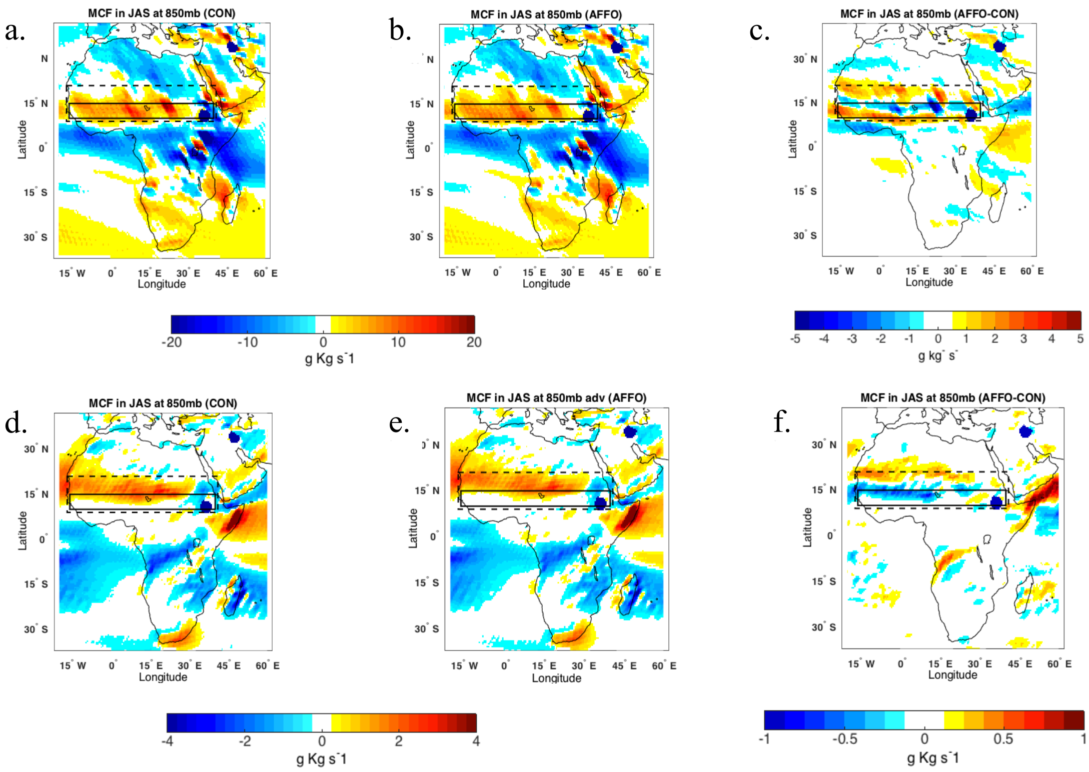

Figure S6. Model simulated seasonal mean (JAS) during the period 1998 -2012, of Moist Flux Convergence (MFC) (a)-(c) convergence/divergence (positive/negative) over Africa for the 850 mb, (a) CON-2d, (b) AFFO-2d-S and (c) AFFO-2d-S – CON-2d, and (d)-(f) advection in to/out of the region (positive/negative). Solid lines represent the afforested area [10^0-15^0 N, 16^0 W-40^0 E], and the dash lines cover the footprint area [10^0-20^0 N, 16^0 W-40^0 E]. Units in [10^3 g kg^{-1} s^{-1}]. Maps in this figure were created using Matlab R2014b (http://www.mathworks.com/).

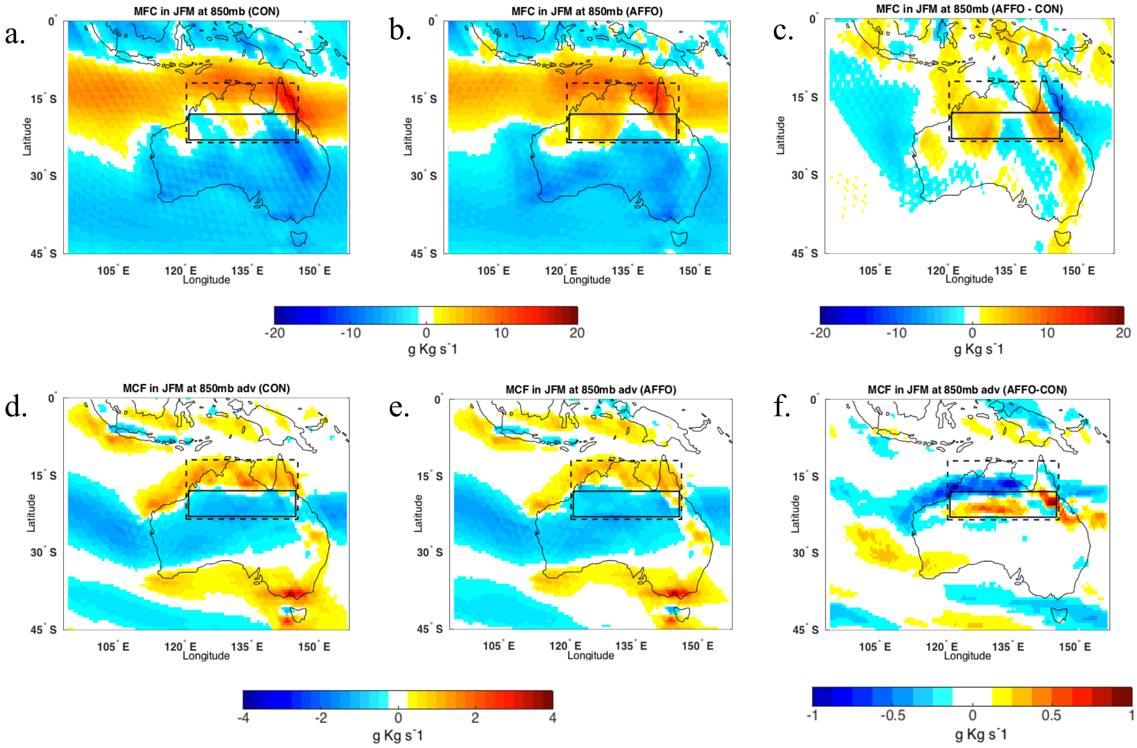

Figure S7. Model simulated seasonal mean (JFM) during the period 1998 -2012, of Moist Flux Convergence (MFC) (a)-(c) convergence/divergence (positive/negative) over N-Aust for the 850 mb, (a) CON-2d, (b) AFFO-2d-NA and (c) AFFO-2d-NA – CON-2d, and (d)-(f) advection in to/out of the region (positive/negative). Solid lines represent the afforested area [17^0-22^0 S, 122^0 -146^0 E], and the dash lines cover the footprint area [12^0-22^0 S, 122^0 -146^0 E]. Units in [10^3 g kg^{-1} s^{-1}]. Maps in this figure were created using Matlab R2014b (http://www.mathworks.com/).

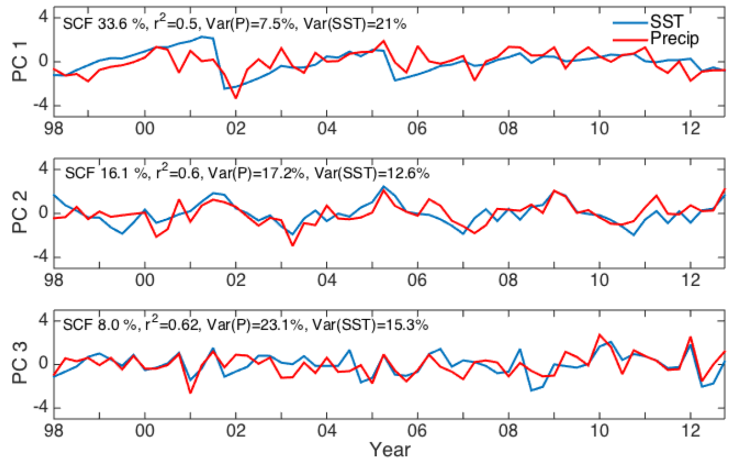

Figure S8. The non-dimensional normalized SST and precipitation principal component for the first three SST and precipitation modes over the Sahel.

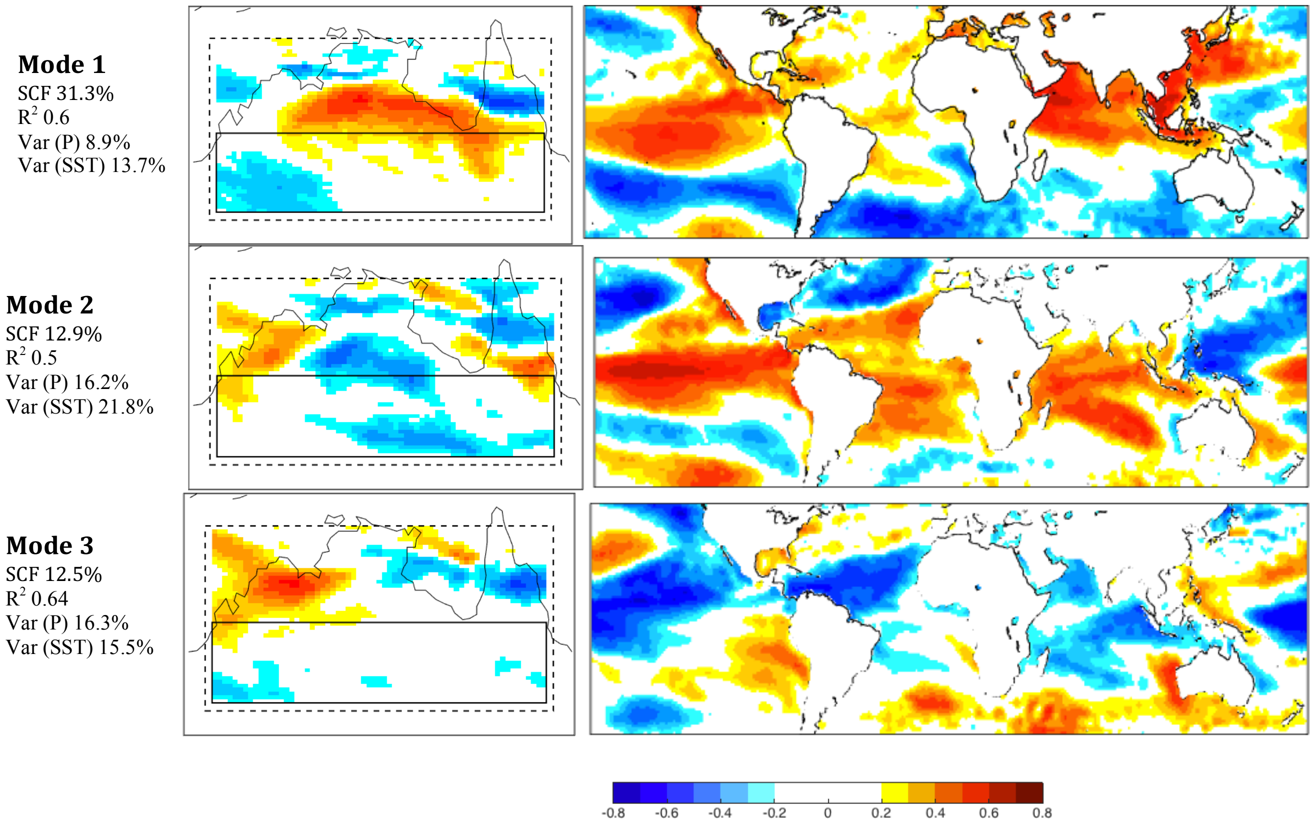

Figure S9. Leading mode derived from the MCA analysis of December to March (DJFM) precipitation (P) and sea surface temperature (SST) anomalies of AFFO-2d-NA– CON-2d simulations from 1998 to 2012: P (left) and SST (right). In the P modes the dashed line represents the areas of the footprint (North Australia); solid lines represent the afforested area. The SST modes represent the ocean basin area associated with the precipitation pattern over North Australia: Mode 1 and 2: combination of mainly ENSO and IOD, Mode 3: warm SST over west and south Australia. The square covariance fraction (SCF) of each mode, the correlation (R^2) between the MCA of the P and SST in the modes and the fraction of the variance (Var) of the given P and SST modes are presented. Maps in this figure were created using Matlab R2014b (http://www.mathworks.com/).

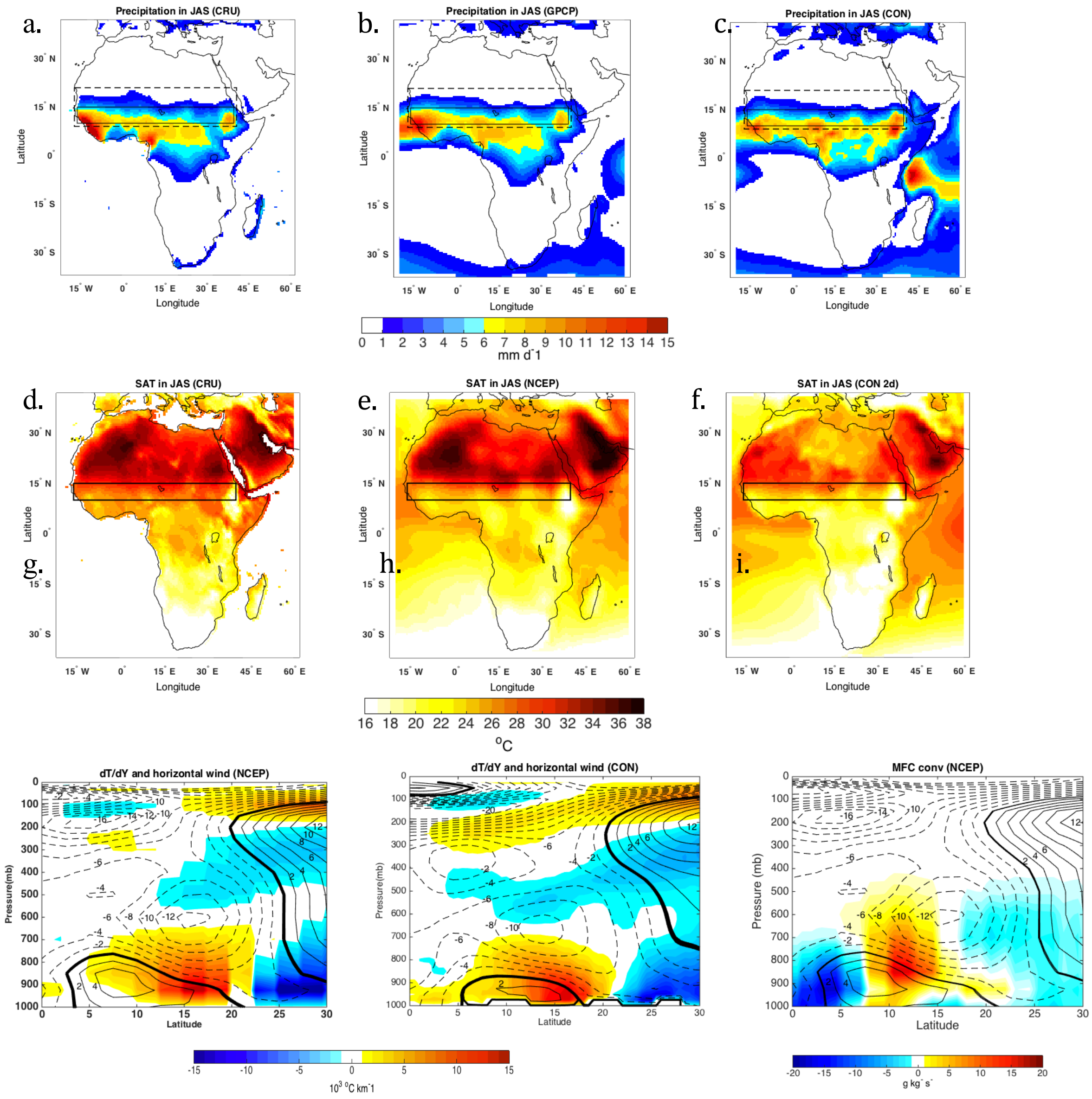

Figure S10: Observation and model simulated over Africa for July-September (JAS) mean between the years 1998-2012. Precipitation: (a) CRU, (b) GPCP and (c) CON-2d. SAT: (d) CRU, (e) NCEP and (f) CON-2d. Zonally (10^0W- 15^0W) averaged meridional temperature gradient dT/dy [10^3 ^{0}C km^{-1}] and zonal wind [m s^{-1}]: (g) NCEP, (h) CON-2d. The total Moisture Flux Convergence (MFC) convergence/divergence (positive/negative) with zonal wind (m s^{-1}): (i) NCEP, Solid lines and dash line represents easterly and westerly winds intensity respectively, Units in [10^3 g kg^{-1} s^{-1}]. The Afforested area [10^0-15^0N, 16^0 W-40^0 E] indicates as solid line and the dash lines cover the footprint area [10^0-20^0N, 16^0 W-40^0 E]. Maps in this figure were created using Matlab R2014b (http://www.mathworks.com/).

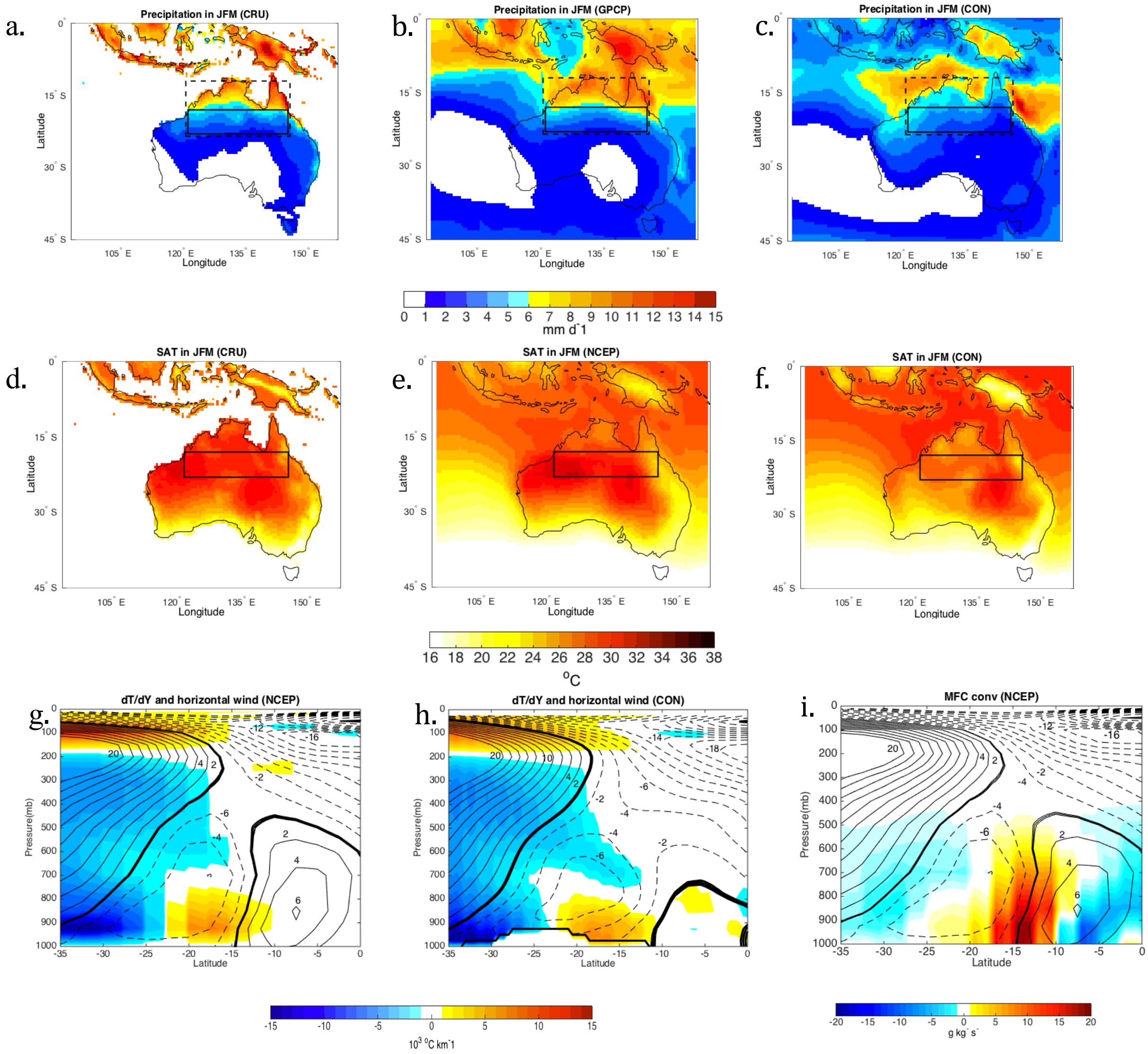

Figure S11: Observation and model simulated over N-Aust for January-March (JFM) mean between the years 1998-2012. Precipitation: (a) CRU, (b) GPCP and (c) CON-2d. SAT: (d) CRU, (e) NCEP and (f) CON-2d. Zonally (130^0-135^0E) averaged meridional temperature gradient dT/dy [10^3 ^{0}C km^{-1}] and zonal wind [m s^{-1}]: (g) NCEP, (h) CON-2d. The total Moisture Flux Convergence (MFC) convergence/divergence (positive/negative) with zonal wind (m s^{-1}): (i) NCEP, Solid lines and dash line represents easterly and westerly winds intensity respectively, Units in [10^3 g kg^{-1} s^{-1}]. The Afforested area [17^0-22^0 S, 122^0 -146^0 E] indicates as solid line and the dash lines cover the footprint area [12^0-22^0 S, 122^0 -146^0 E]. Maps in this figure were created using Matlab R2014b (http://www.mathworks.com/).

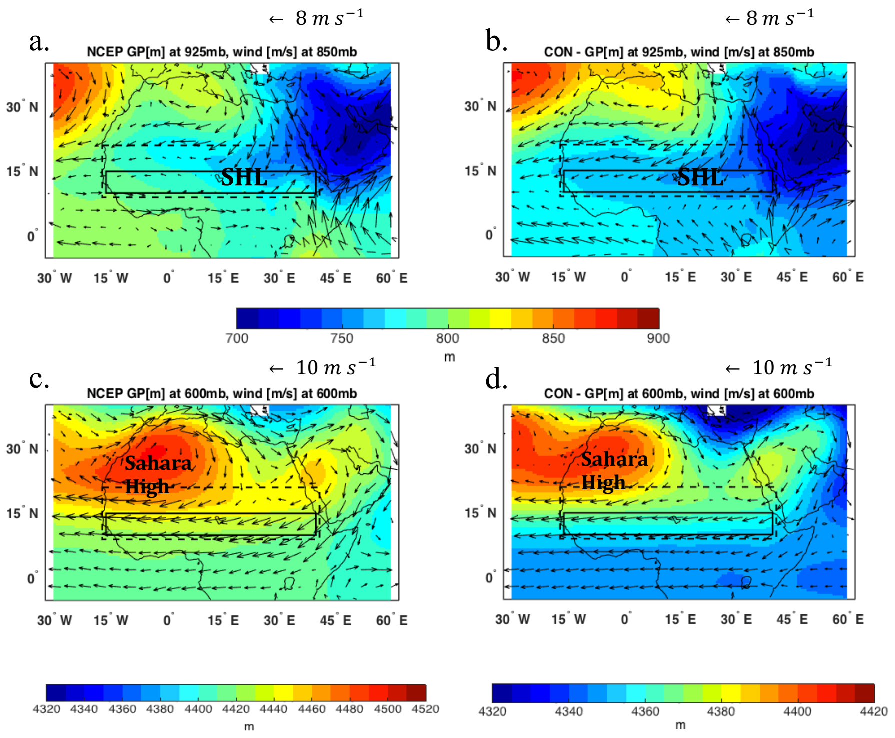

Figure S12. Geopotential height (GP) and total wind vectors [m s^{-1}] of NCEP and CON-2d in JAS during the period 1998-2012. (a) – (b) : GP 925 mb and wind at 850 mb, (c) – (d) : GP and wind at 600 mb. Contour interval is 10[m]. Maps in this figure were created using Matlab R2014b (http://www.mathworks.com/).

a. b.

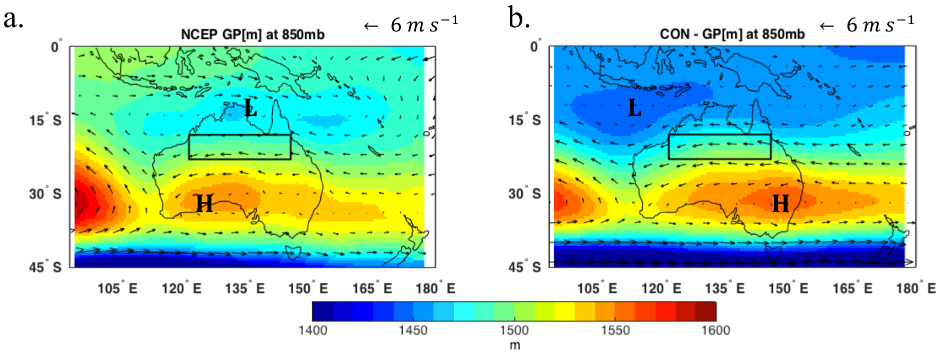

Figure S13. Geopotential height (GP) and total wind vectors [m s^{-1}] at 850 mb of (a) NCEP and (b) CON-2d in JFM during the period 1998-2012, Contour interval is 10[m]. Maps in this figure were created using Matlab R2014b (http://www.mathworks.com/).
